# Synergistic Antiviral Activity of Pamapimod and Pioglitazone against SARS-CoV-2 and Its Variants of Concern

**DOI:** 10.3390/ijms23126830

**Published:** 2022-06-20

**Authors:** Christian Setz, Maximilian Große, Janina Auth, Maria Fröba, Pia Rauch, Alexander Bausch, Matthew Wright, Ulrich Schubert

**Affiliations:** 1 Institute of Virology, Friedrich-Alexander University Erlangen-Nürnberg (FAU), 91054 Erlangen, Germany; christian.setz@uk-erlangen.de (C.S.); maximilian.grosse@uk-erlangen.de (M.G.); janina.auth@fau.de (J.A.); maria.carolin.froeba@fau.de (M.F.); pia.rauch@uk-erlangen.de (P.R.); 2 Kinarus Therapeutics AG, 4057 Basel, Switzerland; alexander.bausch@kinarus.com

**Keywords:** COVID-19, SARS-CoV-2, coronavirus, variants of concern, virus variants, pioglitazone, pamapimod, p38-inhibitor, PPRy-agonist, cellular targets

## Abstract

The SARS-CoV-2 pandemic remains a major public health threat, especially due to newly emerging SARS-CoV-2 Variants of Concern (VoCs), which are more efficiently transmitted, more virulent, and more able to escape naturally acquired and vaccine-induced immunity. Recently, the protease inhibitor Paxlovid^®^ and the polymerase inhibitor molnupiravir, both targeting mutant-prone viral components, were approved for high-risk COVID-19 patients. Nevertheless, effective therapeutics to treat COVID-19 are urgently needed, especially small molecules acting independently of VoCs and targeting genetically stable cellular pathways which are crucial for viral replication. Pamapimod is a selective inhibitor of p38 Mitogen-Activated Protein Kinase alpha (p38 MAPKα) that has been extensively clinically evaluated for the treatment of rheumatoid arthritis. Signaling via p38 has recently been described as a key pathway for the replication of SARS-CoV-2. Here, we reveal that the combination of pamapimod with pioglitazone, an anti-inflammatory and approved drug for the treatment of type 2 diabetes, possesses potent and synergistic activity to inhibit SARS-CoV-2 replication in vitro. Both drugs showed similar antiviral potency across several cultured cell types and similar antiviral activity against SARS-CoV-2 Wuhan type, and the VoCs Alpha, Beta, Gamma, Delta, and Omicron. These data support the combination of pamapimod and pioglitazone as a potential therapy to reduce duration and severity of disease in COVID-19 patients, an assumption currently evaluated in an ongoing phase II clinical study.

## 1. Introduction

Coronavirus disease 2019 (COVID-19) caused by the severe acute respiratory syndrome coronavirus 2 (SARS-CoV-2) can cause both short and long-term complications, including need for respiratory support and persistent cardiovascular complications. To date, the COVID-19 pandemic has resulted in over 500 million global cases and over 6 million deaths [1]. The emergence and spread of SARS-CoV-2 variants has become a major threat to public health. In particular, the so-called “Variants of Concern” (VoCs) have the potential to evade vaccine- or infection-induced antiviral immune responses [2,3].

Mutations in SARS-CoV-2 VoCs generally affect the infectivity, transmissibility, or pathogenicity of the virus. Mutations have been identified primarily in the spike glycoprotein, with the majority affecting interaction with host receptors [4,5,6,7]. The VoCs discovered to date include SARS-CoV-2 Alpha [8], SARS-CoV-2 Beta [9], SARS-CoV-2 Gamma [10], as well as SARS-CoV-2 Delta [11]. These VoCs were reported to show higher transmissibility and infectivity [4,12,13,14,15,16]. In November 2021, the emergence of SARS-CoV-2 Omicron as a fifth VoC was first detected in South Africa [17]. This variant is currently the predominant variant in most countries. Omicron contains several deletions, insertions, and mutations, overlapping with those of other VoCs that are known to lead to higher transmissibility, viral affinity, and antibody escape [18]. Overall, a total of 60 mutations have been described, with over 30 mutations located in the spike glycoprotein [19]. Very recently, 247 human receptor binding domain (RBD) neutralizing antibodies were characterized, demonstrating that >85% were ineffective against the Omicron VoC [20]. In light of the continuing COVID-19 pandemic, the development of broadly effective prophylactic and therapeutic countermeasures remains of utmost importance.

Recently, first specific antiviral small molecule drugs have received emergency use authorization [21]. Nirmatrelvir, an inhibitor of the 3-Chymotrypsin-like protease of SARS-CoV-2 combined with Ritonavir, a small molecular originally developed as an HIV-1-protease inhibitor, were distributed under the label Paxlovid^®^ and approved for high-risk COVID-19 patients [22,23]. Moreover, molnupiravir targeting the RNA-dependent RNA-Polymerase of SARS-CoV-2 has an emergency approval for high-risk COVID-19 patients in some countries, e.g., USA and Japan, but not in the EU [24]. However, these small molecules target mutation-prone viral components, leading to high risk of the development of drug-resistance, especially when administered as monotherapy. As host cell targets are almost invariably crucial for viral replication, regardless of viral variants, inhibitors of targets in key host cell pathways should possess broad and durable antiviral activity [25].

The role of p38 MAPK has recently been revealed to be a key player in SARS-CoV-2 replication and inflammatory responses. SARS-CoV-2 binds and downregulates angiotensin-converting enzyme 2 (ACE2) as it enters the cell [26]. ACE2 modulates the function of angiotensin II (Ang II). A number of reports indicate that the p38 MAPK pathway is important in Ang II signaling [27]. Ang II increases blood pressure and inflammation, leading to tissue injury, particularly in alveoli [28,29]. The interaction between the SARS-CoV-2 spike protein reduces the inhibition of Ang II, leading to tissue damage often observed in COVID-19 patients. Ang 1-7 activation of the Mas receptor reduces p38 MAPK activation, resulting in reduced inflammation [30]. The loss of ACE2 activity upon viral entry may, therefore, allow Ang II mediated activation of p38 in the lungs and heart, resulting in excessive inflammation. Moreover, a positive feedback loop by upregulation of a disintegrin and metalloprotease 17 (ADAM17), which cleaves the ACE2 ectodomain, may further reduce protection afforded by local ACE2 [31]. Based on these data, p38 MAPK inhibitors previously developed for other indications may be repurposed as a potential therapy for COVID-19.

Here, we investigated the efficacy of pamapimod (PAM), a potent and selective inhibitor of p38 MAPKα, previously developed for the treatment of rheumatoid arthritis, on SARS-CoV-2 replication in vitro [32,33]. As previous data have suggested substantial crosstalk between the p38 MAPK and peroxisome proliferator-activated receptors γ (PPARγ) pathways [34], we have also investigated the combination of PAM with pioglitazone (Pio). Pio belongs to the family of thiazolidinedione (TZD) drugs that are used to lower glucose and HbA1C in type 2 diabetic patients [35]. Pio also has broad anti-inflammatory activity, exemplified by its ability to significantly reduce interleukin-6 (IL-6) and tumor necrosis factor α (TNFα) in insulin resistant individuals without manifest hyperglycemia matched for age, gender, and adiposity [36]. Moreover, Pio has also been shown to inhibit monocyte gene and protein expression of IL-1b, IL-6, and IL-8 and lymphocyte IL-2, IL-6, and IL-8 [37]. It has also been reported that Pio inhibits the secretion of pro-inflammatory cytokines (e.g., IL-1b, IL-6, and IL-8) and can increase expression of anti-inflammatory cytokines (e.g., IL-4 and IL-10) in astrocytes stimulated with lipopolysaccharide [38].

Our data show that PAM potently inhibited the replication of SARS-CoV-2 with IC_50_ and IC_90_ values of approximately 100 nM and 3 µM, respectively. PAM demonstrated similar antiviral activity against the SARS-CoV-2 Wuhan type and all VoCs across several cell lines. We also showed that treatment with Pio strongly reduced the release of SARS-CoV-2 progeny virions with an IC_50_ of approximately 800 nM and IC_90_ of ~10 µM. The combination of the two drugs demonstrated synergistic activity against the original SARS-CoV-2 strain, as well as against the most problematic VoCs, Delta and Omicron. Our data suggest that combined treatment with PAM and Pio should be considered as a potentially effective treatment to reduce severity and time to recovery in COVID-19 patients.

## 2. Results

### 2.1. The p38-Specific Inhibitor Pamapimod (PAM) Exhibits Efficient Antiviral Activity against SARS-CoV-2 in Various Cell Lines

**Figure 1:**
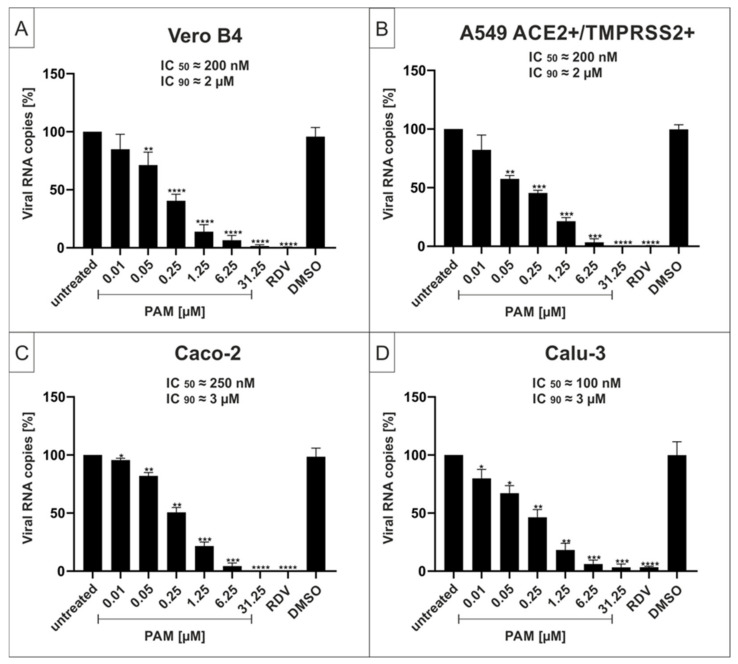
Pamapimod (PAM) inhibits replication of SARS-CoV-2 Wuhan type with comparable antiviral efficacy in various cell lines. Vero B4 (**A**), A549-ACE2+/TMPRSS2+ (**B**), Caco-2 (**C**), and Calu-3 cells (**D**) were infected with the clinical isolate SARS-CoV-2_PR-1_ at a MOI of 2 × 10^−2^. One hour after infection and removal of input virus, cells were treated with the indicated concentrations of PAM; 1 µM Remdesivir (RDV) was included as a positive control, and DMSO was added as a solvent control in the same amount as to the highest used amount of PAM (31.25 µM). Cell culture supernatants were harvested at 3 dpi. The virions were purified and analyzed by qRT-PCR. Data represent means of three independent experiments ± standard deviation (* *p* < 0.046, ** *p* < 0.0086, *** *p* < 0.0006, and **** *p* < 0.0001) using a One sample t test, where each value is compared to the untreated control.

In order to determine whether the p38 MAPKα-specific inhibitor, PAM, exhibits antiviral activity against SARS-CoV-2, Vero cells (African green monkey kidney cells), human Caco-2 colon carcinoma-derived epithelial cells [39], A549 cells, which were generated from a human lung adenocarcinoma and stably express both the ACE2 receptor and transmembrane protease serine subtype 2 (TMPRSS2) [40], and Calu-3 human lung cells (the most extensively studied surrogate lung cell infection model that expresses ACE2 and TMPRSS2 endogenously [40]) were infected with SARS-CoV-2 Wuhan type (Figure 1). One hour post infection, different concentrations of PAM were added to the cell cultures. Three days post infection (dpi), cell culture supernatants were harvested, and virus production was analyzed by quantitative RT-PCR (qRT-PCR) (Figure 1).

Treatment with PAM led to strong reduction of virus replication in all infected cell lines. At a concentration of 6.25 µM, PAM almost completely blocked the production of progeny virions. The IC_50_ values were similar with small variations between ~100 nM in Calu-3 (Figure 1D), ~200 nM in Vero B4 and A549-ACE2+/TMPRSS2+ (Figure 1A,B) and ~250 nM in Caco-2 cells (Figure 1C). The IC_90_ values ranged between 2–3 µM and thus were almost identical among all investigated cell lines (Figure 1).

In addition, and to confirm the results obtained from the qRT-PCR analysis of the cell culture supernatants, Calu-3 cells were infected with the wildtype isolate SARS-CoV-2_PR-1_ at the same MOI (Figure 1) and subsequently treated with different concentrations of PAM for 30 h. Cells were fixed and further stained with a SARS-CoV-2 nucleoprotein (NP)-specific antibody. RDV was used as a positive control at 1 µM (Figure S1, see Supplementary Materials). PAM effectively suppressed SARS-CoV-2 replication in a dose-dependent manner within the infected cells confirming the qRT-PCR data (Figure S1).

**Figure 2:**
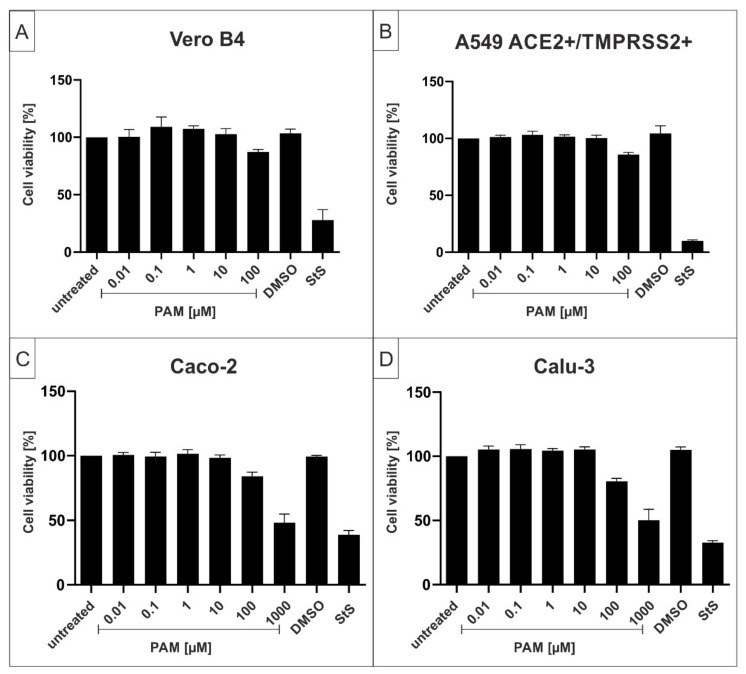
Influence of PAM on the cell viability of Vero B4 (**A**), A549-ACE2+/TMPRSS2+ (**B**), Caco-2 (**C**), and Calu-3 cells (**D**). Following treatment with different concentrations of PAM (PAM concentrations are indicated at the *x*-axis) for three days, the influence on cell viability was measured by water-soluble tetrazolium salt (WST)-1 assay. Bars represent means of three independent experiments ± SD. Staurosprine (StS, 1 µM) was used as a positive control. DMSO as solvent control was added in the same amount as in the highest concentration of PAM (100 µM (**A**,**B**); 1000 µM (**C**,**D**)).

To control for potential unspecific effects of PAM treatment on cell viability, water-soluble tetrazolium salt (WST)-1 assays were performed in uninfected Vero B4, A549-ACE2/TMPRSS2+, Caco-2, or Calu-3 cells under otherwise identical conditions as the virus infection experiments. The results, summarized in Figure 2, demonstrate that treatment with PAM at concentrations up to 100 µg/mL, several-fold higher than the concentrations that fully suppress SARS-CoV-2 replication in all tested settings, had no impact on cell viability in all cell types (Figure 2). The TD_50_ values for PAM were ~1000 µM in Caco-2 and Calu-3 cells. Staurosporine (StS) was used as a positive control at a concentration of 1 µM. DMSO as a solvent control was added in the same amount as the respective highest concentration of PAM.

### 2.2. The PPARγ-agonist Pioglitazone (Pio) Exhibits Efficient Antiviral Activity against SARS-CoV-2 in Various Cell Lines

**Figure 3:**
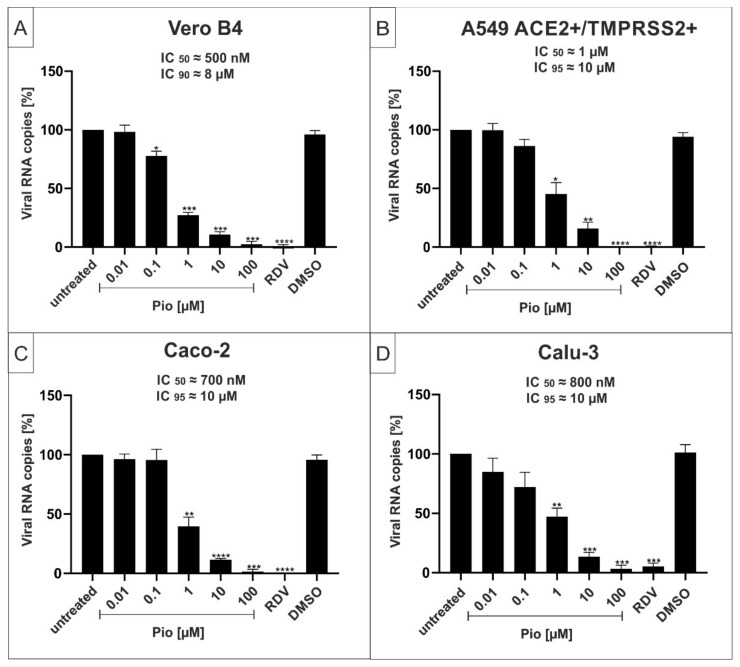
Pioglitazone (Pio) inhibits replication of SARS-CoV-2 Wuhan type with comparable antiviral efficacy in various cell lines. Vero B4 (**A**), A549-ACE2+/TMPRSS2+ (**B**), Caco-2 (**C**), and Calu-3 cells (**D**) were infected with SARS-CoV-2_PR-1_ at a MOI of 2 × 10^−2^. One hour after infection and removal of input virus, cells were treated with the indicated concentrations of Pio; 1 µM RDV was included as a positive control, and DMSO as a solvent control, and these were tested at the same concentration as used in the highest concentration of Pio (100 µM). Cell culture supernatants were harvested at 3 dpi. The virions were purified and analyzed by qRT-PCR. Data represent means of three independent experiments ± standard deviation (* *p* < 0.01, ** *p* < 0.0058, *** *p* < 0.0006, and **** *p* < 0.0001) using a One sample t test, where each value is compared to the untreated control.

Next, we performed similar experiments to assess the potential antiviral effects of the PPARγ-agonist Pio against the SARS-CoV-2 Wuhan type in various cell lines. Similar to the experiments conducted in Figure 1, viral infections were performed in Vero B4, A549-ACE2/TMPRSS2+, Caco-2, or Calu-3 cells with SARS-CoV-2 Wuhan type and treated with increasing concentrations of Pio. Cell culture supernatants were harvested, and virus production was analyzed by qRT-PCR (Figure 3).

Similar to the effects of PAM (Figure 1), treatment with Pio led to a strong reduction of virus replication in all infected cell lines. The IC_50_ values were ~500 nM in Vero B4 (Figure 3A), ~700 nM in Caco-2 (Figure 3C), ~800 nM in Calu-3 (Figure 3D), and ~1 µM in A549-ACE2+/TMPRSS2+ (Figure 3B). The IC_90_ values varied between approximately 8–10 µM and were, thus, comparable in all investigated cell lines (Figure 3).

To exclude potential unspecific effects of Pio treatment on cell viability, WST-1 assays were performed in uninfected Vero B4, A549-ACE2/TMPRSS2+, Caco-2, or Calu-3 cells under otherwise identical conditions as for the infection experiments.

**Figure 4:**
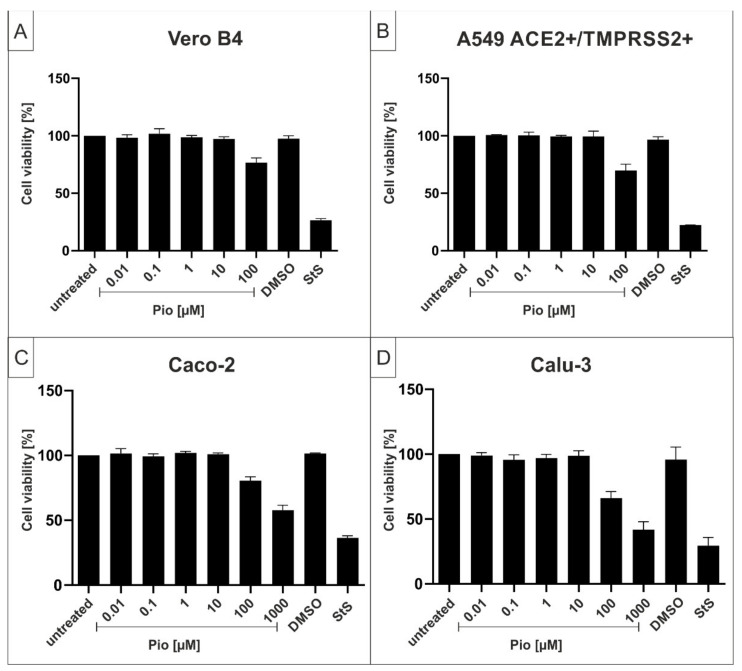
Influence of Pio on the cell viability of Vero B4 (**A**), A549-ACE2+/TMPRSS2+ (**B**), Caco-2 (**C**), and Calu-3 cells (**D**). Following treatment with different concentrations of Pio (Pio concentrations are indicated on the *x*-axis) for three days, the influence on cell viability was measured by WST-1 assay. Bars represent means of three independent experiments ± SD. StS (1 µM) was used as a positive control. DMSO as a solvent control, was tested at the same concentration as used in the highest concentration of Pio (100 µM (**A**,**B**); 1000 µM (**C**,**D**)).

Treatment with Pio at concentrations up to ~80 µM, which completely suppresses SARS-CoV-2 replication in all test settings, had no impact on cell viability in all cell types (Figure 4). The TD_50_ values for Pio were between ~500–1000 µM in Caco-2 and Calu-3 cells (Figure 4C,D).

### 2.3. Both PAM and Pio Exhibit Comparable Antiviral Activity against all SARS-CoV-2 Variants of Concern

**Figure 5:**
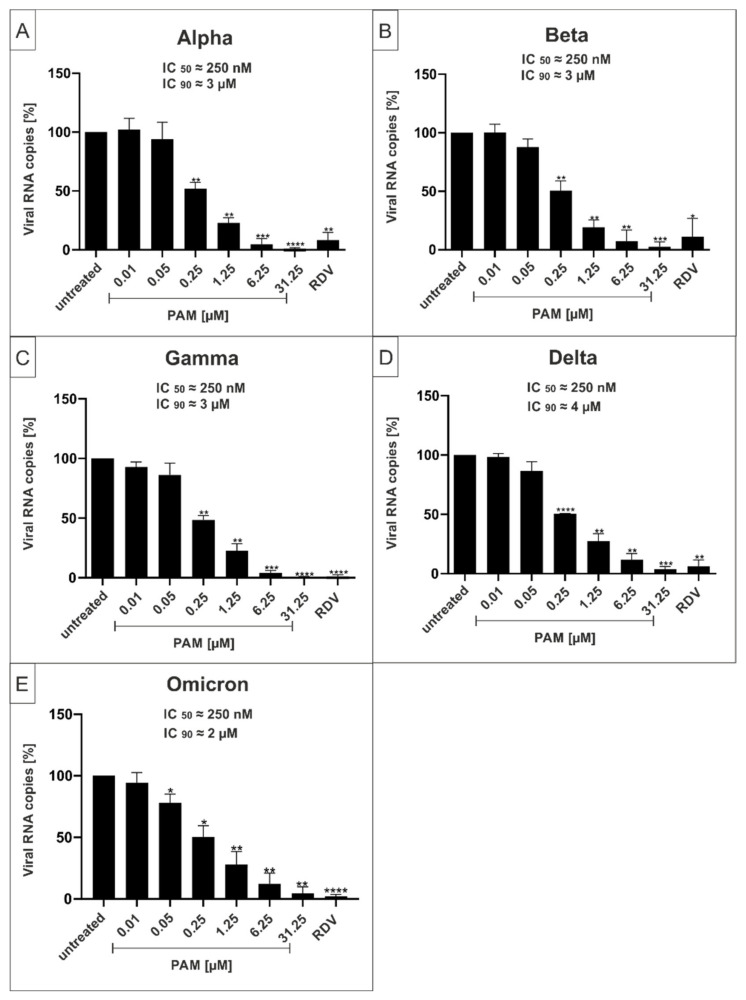
PAM inhibits replication of the SARS-CoV-2 Variants of Concern (VoCs) Alpha, Beta, Gamma, Delta, and Omicron with comparable antiviral efficacy. Calu-3 cells were infected with clinical isolates of the SARS-CoV-2 VoCs Alpha (**A**), Beta (**B**), Gamma (**C**), Delta (**D**), and Omicron (**E**) at a MOI of 2 × 10^−2^. One hour after infection and removal of input virus, cells were treated with the indicated concentrations of PAM; 1 µM RDV was included as a positive control. Cell culture supernatants were harvested at 3 dpi. The virions were purified and analyzed by qRT-PCR. Data represent means of three independent experiments ± standard deviation (* *p* < 0.05, ** *p* < 0.0089, *** *p* < 0.0008, and **** *p* < 0.0001) using a One sample t test, where each value is compared to the untreated control.

**Figure 6:**
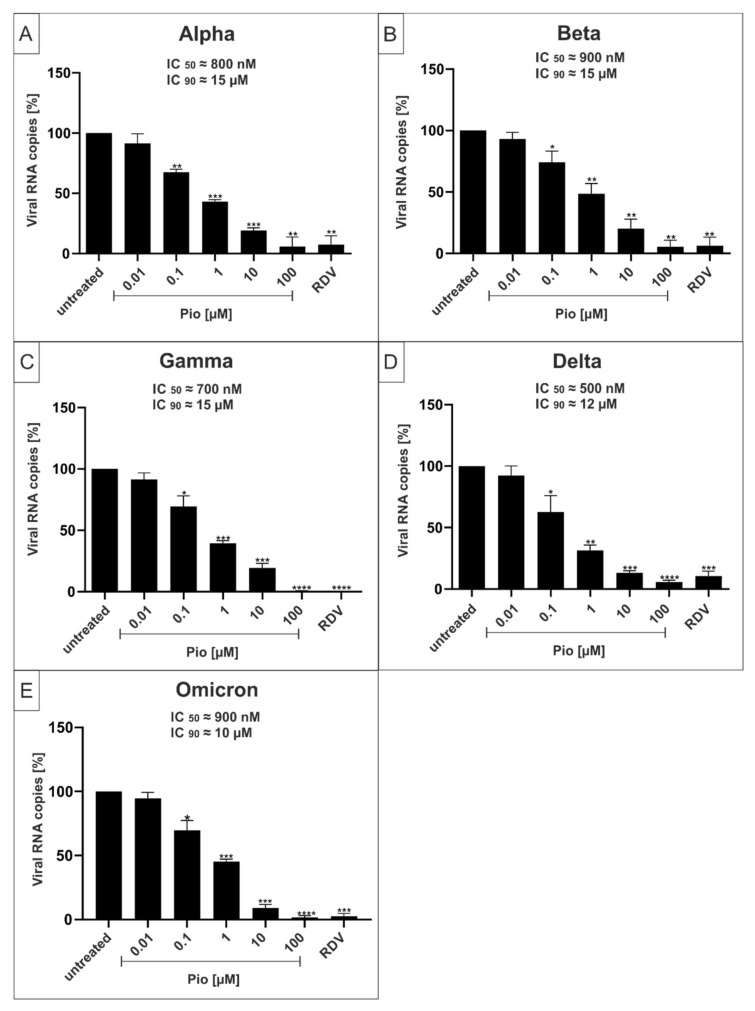
Pio inhibits replication of the SARS-CoV-2 VoCs Alpha, Beta, Gamma, Delta and Omicron with comparable antiviral efficacy. Calu-3 cells were infected with the SARS-CoV-2 VoCs Alpha (**A**), Beta (**B**), Gamma (**C**), Delta (**D**), and Omicron (**E**) at a MOI of 2 × 10^−2^. One hour after infection and removal of input virus, cells were treated with the indicated concentrations of Pio; 1 µM RDV was included as a positive control. Cell culture supernatants were harvested at 3 dpi. The virions were purified and analyzed by qRT-PCR. Data represent means of three independent experiments ± standard deviation (* *p* < 0.04, ** *p* < 0.0089, *** *p* < 0.0003, and **** *p* < 0.0001) using a One sample *t* test, where each value is compared to the untreated control.

In order to determine whether PAM and Pio exhibit a comparable, broad antiviral activity against all described VoCs of SARS-CoV-2, Calu-3 human lung cells were infected with the VoCs Alpha, Beta, Gamma, Delta, and Omicron (Figure 5 and Figure 6). One hour post infection, different concentrations of PAM (Figure 5) or Pio (Figure 6) were added to the cells. Three dpi, cell culture supernatants were harvested, and virus production was analyzed by qRT-PCR (Figure 5 and Figure 6).

Treatment with PAM or Pio led to a strong dose-dependent reduction of virus replication that occurred with comparable efficacy for all VoCs (Figure 5 and Figure 6). For PAM, the IC_50_ was approx. 250 nM for all VoCs, which is also in a comparable range as shown for the Wuhan type (~100 nM; Figure 1D). The IC_90_ slightly varies between 3 and 4 µM (Figure 5), which is lower than demonstrated for the Wuhan type (~10 µM; Figure 1D).

For Pio, the IC_50_ values varies between ~500 nM for Delta (Figure 6D), ~700 nM for Gamma and Omicron (Figure 6C), ~800 nM for Alpha (Figure 6A), and ~900 nM for Beta (Figure 6B). This was in a similar range as shown for the Wuhan type (~800 nM; Figure 3D). The IC_90_ values varied between 12 and 15 µM (Figure 6), which is slightly higher than demonstrated for the Wuhan type (~10 µM; Figure 3D). The IC_50_ and IC_90_ values for PAM and Pio following infection with the Wuhan type and respective VoCs are summarized in Table 1. In comparison, the published IC_50_ and IC_90_ values for the control Remdesivir are 600 nM and 1.28 µM in Calu-3, or 1.49 µM and 3 µM in Vero E6 cells [41].

In conclusion, we could demonstrate that both PAM and Pio possess comparable strong antiviral activity against SARS-CoV-2 independent of the variant, pointing towards a central role of both p38 MAPK and PPARγ in the cellular replication cycle of SARS-CoV-2.

### 2.4. Combination Treatment with PAM and Pio Exhibits Synergistic Antiviral Activity against SARS-CoV-2 Wuhan Type and the VoCs Delta and Omicron

**Figure 7:**
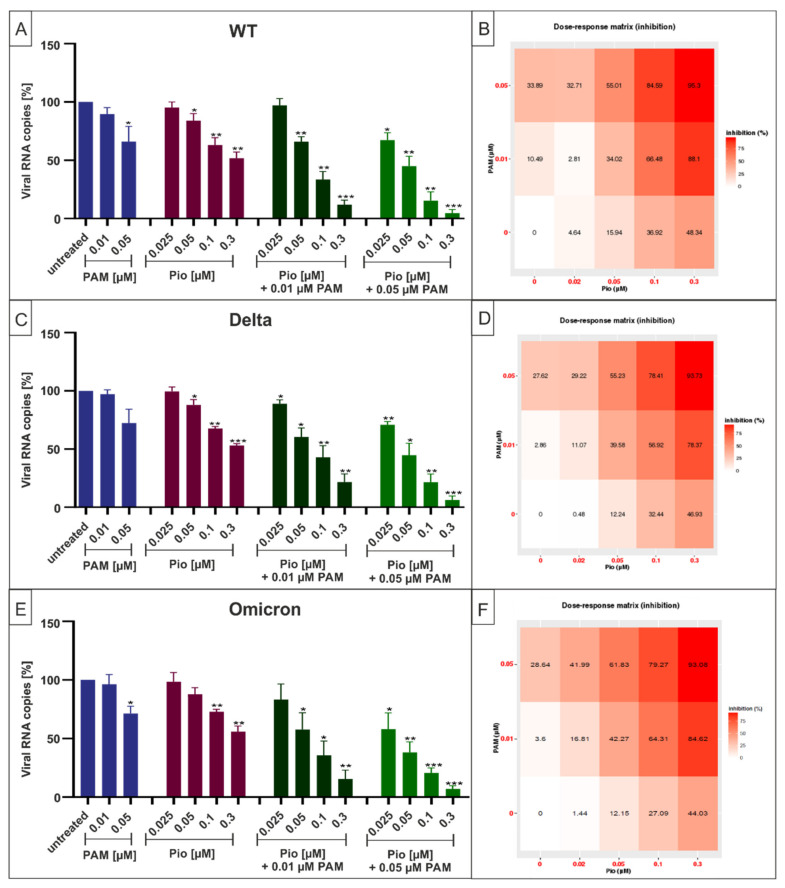
Antiviral activity of the combinatory treatment of PAM with Pio following infection with SARS-CoV-2 Wuhan type, Delta, and Omicron. (**A**,**C**,**E**) Calu-3 cells were infected with SARS-CoV-2_PR-1_ (**A**), SARS-CoV-2 Delta (**C**), and SARS-CoV-2_OM_ (**E**) at a MOI of 2 × 10^−2^. One hour after infection and removal of input virus, cells were treated with indicated concentrations of PAM (blue), Pio (red), or the combinatory treatment of PAM and Pio (green). Cell culture supernatants were harvested at 3 dpi. The virions were purified and analyzed by qRT-PCR. Data represent means of three independent experiments ± standard deviation (* *p* ≤ 0.05, ** *p* < 0.005 and *** *p* < 0.001) using a One sample t test, where each value is compared to the untreated control. (**B**,**D**,**F**) percent of the inhibition of viral replication following combined treatment with PAM and Pio and infection with SARS-CoV-2_PR-1_ (**B**), SARS-CoV-2 Delta (**D**), and SARS-CoV-2_OM_ (**F**). The tables were created using the open-source and free web application SynergyFinder [42].

As previous data have revealed extensive crosstalk between the p38MAPK and PPARγ pathways [34], we next evaluated whether or not treatment with the combination of PAM and Pio may have additive or synergistic antiviral activity against SARS-CoV-2 and its VoCs. Therefore, Calu-3 cells were infected with SARS-CoV-2 Wuhan Type, Delta, or Omicron. One hour post infection, different concentrations of PAM or Pio, alone or in combination, were added to the cell cultures (Figure 7). Three dpi cell culture supernatants were harvested, and virus production was analyzed by qRT-PCR (Figure 7).

Following treatment with increasing amounts of Pio (25–300 nM) in combination with 10 nM of PAM, a concentration that exhibited non-significant effect on virus replication (Figure 1 and Figure 5), significant and dose-dependent reduction in replication capacity ranging from 10–88%, from the lowest to highest concentration of Pio was observed for SARS-CoV-2 Wuhan type, as well as the VOCs Delta and Omicron (Figure 7). Treatment with the identical concentration series of Pio in the presence of 50 nM PAM resulted in greater antiviral efficacy, leading to a reduction of viral replication ranging from 27–95%, from the lowest to highest concentration of Pio (Figure 7B,D,F). Further experiments demonstrated comparable reduction in replication of the VoCs Delta and Omicron by the PAM/Pio combination vs. the SARS-CoV-2 Wuhan type.

In contrast, individual treatment with PAM or Pio alone had only minor effects on virus replication at these concentrations. Single treatment with the highest tested concentration of PAM (50 nM) reduced virus replication by max. 33% (Figure 7B), while the highest concentration of Pio (300 nM) reduced viral replication by max. 48% (Figure 7D). However, combination treatment (50 nM PAM and 300 nM Pio) led to almost complete inhibition of viral replication, pointing towards a synergistic antiviral effect.

To evaluate drug combination profiles, synergy scoring is a very important parameter. Thus, we analyzed if the combinational treatment of PAM and Pio exhibits synergistic antiviral activity on SARS-CoV-2 Wuhan type and the VoCs Delta and Omicron, employing the Bliss independence model [43]. This model assumes a stochastic process whereby two small molecules develop their effects independently [42,43]. A Bliss synergy score < −10 means that the two tested substances act antagonistically, a score between −10 and 10 represents additive activity, while a score >10 indicates drug synergism [42].

**Figure 8:**
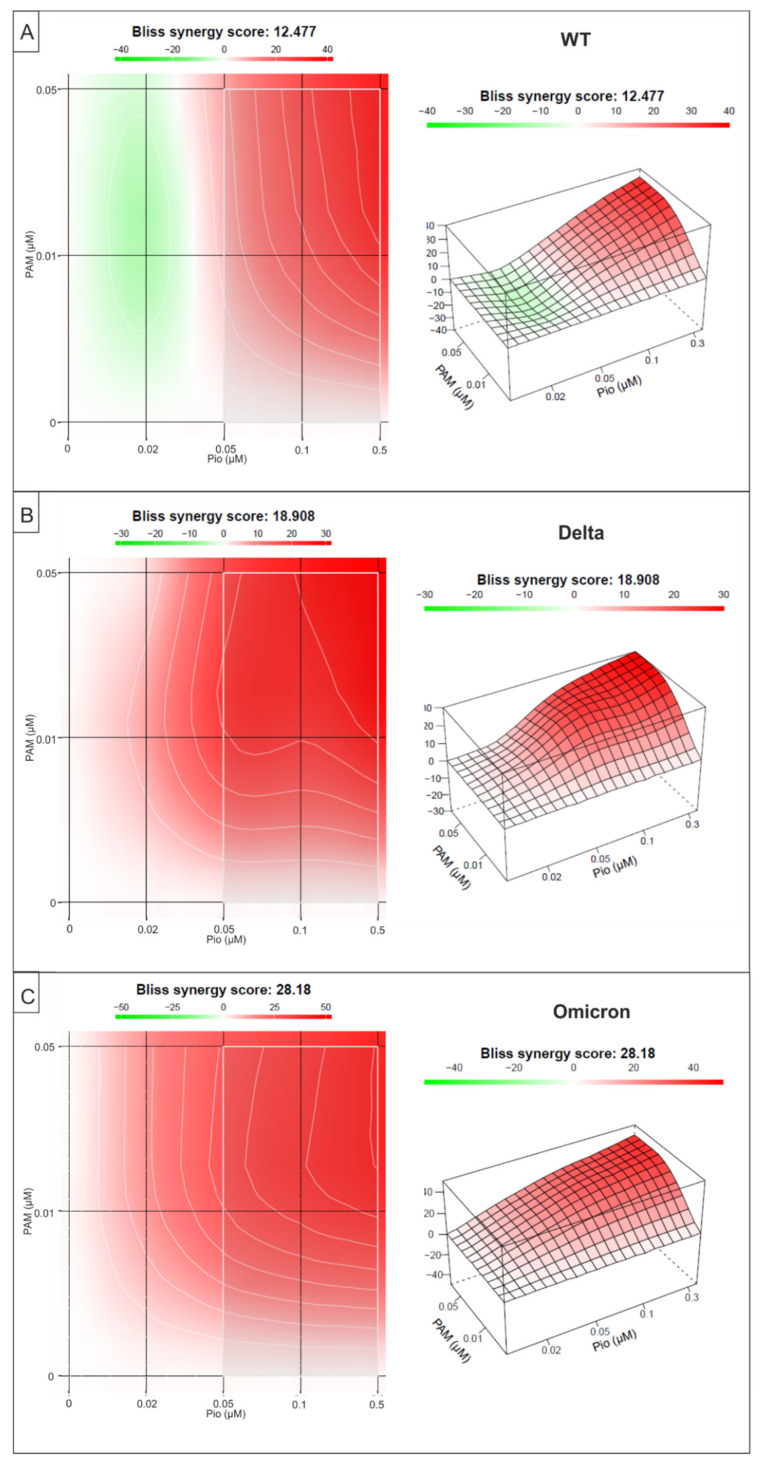
Interaction profile of PAM and Pio for determining the synergy in the inhibition of the replication of SARS-CoV-2 Wuhan type and the VoCs Delta and Omicron. Drug interactions were analyzed using the reference model Bliss independence. The illustrations were created using the open-source and free web application SynergyFinder [42]. The synergy calculations were performed on data derived from the experiments in Calu-3 cells for (**A**) SARS-CoV-2 Wuhan Type, (**B**) Delta, and (**C**) Omicron. The data for each SARS-CoV-2 variant represent means of three independent experiments. A color-coded interaction graphic was used to illustrate the Bliss synergy scores. High synergy scores are colored in red.

The small molecule interaction analysis revealed an overall Bliss synergy score of 12.4 for the combination of PAM and Pio to inhibit replication of SARS-CoV-2 Wuhan type (Figure 8A) and a higher synergy score of 18.8 and 28.1 to inhibit replication of Delta or Omicron, respectively (Figure 8B,C). The highest synergy score was detected following treatment with 50 nM PAM and 10 or 30 nM Pio, independent of the SARS-CoV-2 variant (Figure 8A–C).

In summary, our data clearly show that combination treatment with PAM and Pio exhibits a synergistic effect to inhibit replication of SARS-CoV-2, independently of the virus variant. These data suggest that the combination of the drugs may show substantial efficacy at the doses of the single agents previously employed in human clinical studies (PAM) and for the treatment of type 2 diabetes (Pio) and may also demonstrate clinically meaningful efficacy at lower doses when administered in combination.

## 3. Discussion

Since the beginning of the COVID-19 outbreak in December 2019, caused by SARS-CoV-2, the ongoing pandemic has resulted in a profound health and socioeconomic crisis worldwide. It can be anticipated that, as occurred previously for SARS-CoV and Middle East respiratory syndrome-related coronavirus (MERS-CoV), coronaviruses will continue to evolve the capacity for zoonotic transmission from animals to humans, potentially leading to new threats.

This illustrates the need to remain prepared for future pandemics. Irrespective of the cause, vaccines are valuable but have limitations, particularly in less developed countries where vaccination rates are low. Vaccines, as exemplified by SARS-CoV-2, often suffer from waning effectiveness, and the ever-present chance of emergence of vaccine resistant variants. Society will continue to need new therapeutics that are durably active, safe, cost-effective, and may be stockpiled in preparation for future pandemic threats.

Due to the continuing emergence of new SARS-CoV-2 variants with mutations in the spike protein, the use of antibodies as therapeutics remains a challenge. For the currently predominant VOC Omicron, it has been shown that most of the available human RBD neutralizing antibodies are ineffective [20]. Moreover, treatment with antibodies is expensive and limited primarily to hospitalized patients.

Paxlovid^®^ and Molnupiravir both target viral components which are a million times more mutation-prone in comparison to stable cellular targets, which are crucial for virus replication [44].

The p38 mitogen-activated protein kinases (p38 MAPK) (p38α, p38β, p38γ, and p38δ) play crucial roles in mediating the effector response to environmental stress, pathogenic infection, and pro-inflammatory mediators [45]. Downstream effectors of the pathway include transcription factors and RNA binding proteins that integrate environmental stimuli to regulate inflammatory cytokine production [46,47], cellular proliferation, differentiation, development, and apoptosis [48].

The p38 MAPK pathway has been shown to be important in viral infections. Many viruses, including hepatitis C virus (HCV), influenza virus, enterovirus 71 (EV71), human immunodeficiency virus (HIV), dengue virus (DENV), and hepatitis B virus (HBV) can activate p38 MAP kinases [26,49]. Among other functions, viral p38 MAPK activation induces endocytosis of viral receptors to facilitate viral entry, including promoting endocytosis of ACE2, the cellular receptor for SARS-CoV-2.

SARS-CoV-2 infection upregulates components of the p38 MAPK pathway through the increased phosphorylation of several p38 substrates [50]. The p38 MAPK inhibitor SB203580 demonstrated significant antiviral activity against SARS-CoV-2 in vitro [51]. SB203580 also downregulated the expression of several inflammatory cytokines such as interleukin-6 (IL-6) and TNF-α, and additionally blocked secretion of others including IL-6, CXCL8, CCL20, and CCL2. A small in vivo study in SARS-CoV-2 infected mice showed increased survival in animals treated with the p38 inhibitor SB283580 [51].

PPARs are a family of transcription factors involved in insulin responses including regulation of glycemic control, adipogenesis, and inflammation [52]. Oral Pio, an agonist of PPARγ, is approved as an adjunct to diet and exercise to improve glycemic control in adults with type 2 diabetes mellitus (T2DM) [53]. Diabetes is a significant risk factor for poor outcomes in COVID-19 [54]. A meta-analysis of 78,874 patients hospitalized with COVID-19 revealed that pre-existing diabetes doubled the risk for severe COVID-19 (odds ratio (OR) 2.10) and almost tripled in-hospital mortality (OR 2.68) [55]. A second meta-analysis involving 16,003 subjects also found greater COVID-19 severity with approximately two-fold higher mortality in diabetic vs. non-diabetic patients [56]. A separate retrospective cohort study evaluated the effects of anti-diabetic medications, including Pio, on the incidence of hospital admissions, respiratory complications, and mortality after a COVID-19 diagnosis [57]. The use of Pio was associated with a significant reduction in hospital admissions (20.0% vs. 28.2%; RR 0.71). These data suggest that use of glucose-lowering medications, such as Pio, may improve COVID-19 outcomes for patients with T2DM.

We hypothesized that the combination of a p38 MAPK inhibitor with Pio may have additive or synergistic antiviral activity against SARS-CoV-2 and potentially greater benefit in treatment of COVID-19, particularly in patients with significant comorbidities. Several potential mechanisms support this possibility. Liganded PPARγ may promote nuclear retention of p38 MAPK, reducing its availability to the regulatory upstream Mitogen-activated Protein Kinase Kinase 3 (MKK3) and MKK6 [34]. Potential beneficial interactions may occur due to competition for transcriptional cofactors and substrates. In this regard, PPARγ can negatively regulate gene expression by a ligand-dependent trans repression mechanism, antagonizing, among others, the nuclear factor kappa B (NF-κB) and activator protein 1 (AP-1) pro-inflammatory signaling pathways in immune cells [58].

The combined antiviral effects of PAM and Pio may also be partly mediated by alterations in cellular lipids. Interestingly, monocytes from COVID-19 patients were shown to have increased numbers of lipid droplets compared to those from healthy individuals. In vitro, SARS-CoV-2 infection upregulated PPARγ expression and other key lipid metabolic enzymes [59]. Palmitoylethanolamide (PEA), an endocannabinoid-like lipid and agonist of PPARα was able to exert antiviral activity in vitro against SARS-CoV-2 by reducing virus entry and blocking replication, coincident with a decrease in lipid droplets [60].

Our data demonstrated that PAM and Pio each possess antiviral activity against SARS-CoV-2 Wuhan Type and all VoCs. Notably, the combination also demonstrated synergistic antiviral activity with similar potency against several VoCs. The multiple beneficial effects, including antiviral, anti-inflammatory, and antifibrotic activity, suggest that the combination may provide benefit in all severities of COVID-19 and sequential phases of disease presentation. A Phase 2, double-blinded, randomized, placebo-controlled clinical trial of the combination of PAM and Pio in hospitalized COVID-19 patients is currently ongoing (Eudract no. 2020-005849-16).

## 4. Materials and Methods

### 4.1. Inhibitors

Pamapimod was provided by Kinarus Therapeutics AG and was produced by F Hoffmann La Roche, Basel, Switzerland. Pioglitazone was purchased from MSN Organics Private Ltd. (Hyderabad, India). The substances were both dissolved in dimethyl sulfoxide (DMSO). The stock solutions were stored at −20 °C until use.

### 4.2. Viruses

The “Wuhan type” virus SARS-CoV-2_PR-1_, isolated from a 61-year-old patient, was amplified in Vero B4 cells as described in [61]. The virus strains SARS-CoV-2 Alpha, Beta, Gamma, and Delta were obtained from Michael Schindler (University Hospital, Tübingen). The SARS-CoV-2 Alpha variant (210416_UKv) was generated as described in [62]. SARS-CoV-2 Beta was generated as described in [63]. The Gamma (210504_BRv) and the Delta variant (210601_INv) were isolated from throat swabs collected in May 2021 at the Institute for Medical Virology and Epidemiology of Viral Diseases, University Hospital Tübingen, from PCR-positive patients and generated as described in [64]. To obtain a clinical SARS-CoV-2 Omicron isolate (SARS-CoV-2_OM_), 100 µL of an anonymized residual swap sample of a patient infected with the SARS-CoV-2 Omicron variant were passaged on a confluent monolayer of Caco-2 cells. The integrity of the viral genome and the presence of mutations characteristic for the Omicron variant were confirmed by mutation-specific qRT-PCR (Novaplex™ SARS-CoV-2 Variants VII Assay, Seegene, Düsseldorf, Germany) and Illumina-based next generation sequencing using MiSeq reagent kit v2 on a MiSeq™ instrument (Illumina, San Diego, CA, USA). Sequences were analyzed with CLC Genomics Workbench 21 (Qiagen Aarhus A/S, Aarhus, Denmark). The patient sample has in addition to the usual Omicron mutations an R346K mutation in the spike protein and an I4615V mutation in ORF1ab. Viral titers were determined by an endpoint titration assay. For the generation of new virus stock, a virus containing cell culture supernatant was harvested 72 h post infection (hpi) and passed through a 0.45 µm pore-size filter. Virus stocks were stored at −80 °C until further usage.

### 4.3. Infection Experiments

For infection experiments, cells were inoculated with SARS-CoV-2_PR-1_ (Wuhan type) or the VoCs Alpha, Beta, Gamma, Delta, and SARS-CoV-2_OM_ (multiplicity of infection (MOI): 2 × 10^−2^ for 1 h, washed, and further treated with interventions. Then, 72 hpi, virus-containing cell culture supernatants were incubated for 10 min at 95 °C and finally used for qRT-PCR analysis. For titer determination of SARS-CoV-2 virus stocks, A549-ACE2/TMPRSS2 and Calu-3 cells were infected with serial dilutions of the virus stock over 72 h. Afterwards, cells were fixed (4% PFA), permeabilized (0.5% Triton/PBS), blocked (1% BSA/PBS-T), and finally stained with a SARS-CoV-2 NP antibody (Biozol). Endpoint of virus infection was analyzed via fluorescence microscopy and viral titer was calculated by the method of Reed and Muench [65].

### 4.4. Cell Culture

Calu-3 cells were maintained in Minimal Essential Medium (MEM) containing 20% (*v*/*v*) inactivated fetal calf serum (FCS), 1 mM l-glutamine, 100 U/mL penicillin, and 100 µg/mL streptomycin, and 1 mM sodium pyruvate. A549-cells expressing ACE2 and TMPRSS2 were generated by retroviral transduction as described in [61] and cultivated in RPMI 1640 medium containing 10% (*v*/*v*) inactivated FCS, 2 mM l-glutamine, 100 U/mL penicillin, 100 µg/mL streptomycin, and 100 µg/mL blastomycin. Vero B4 cells were maintained in Dulbecco’s Modified Eagle’s Medium (DMEM) containing 10% (*v*/*v*) inactivated FCS, 2 mM l-glutamine, 100 U/mL penicillin, and 100 µg/mL streptomycin. Caco-2 (human colorectal adenocarcinoma) cells were cultured at 37 °C with 5% CO_2_ in DMEM containing 10% FCS, with 2 mM l-glutamine, 100 µg/mL penicillin-streptomycin, and 1% non-essential amino acids.

### 4.5. Assessment of Cell Viability

Viability of uninfected and treated cells was assessed by the water-soluble tetrazolium salt (WST)-1 assay (Roche, Basel, Switzerland) according to the manufacturer’s instructions. Cells were treated for 72 h with various inhibitors according to the protocols of the infection experiments.

### 4.6. Determination of the Amount of Viral RNA Copies from Released Viruses by qRT-PCR

Viruses were quantified by real-time PCR AgPath-ID One-Step RT-PCR Kit from Ambion (Cat: 4387424) software v2.3 (applied Bioscience). PCR primers were used according to [66]: RdRp_fwd: 5′-GTG-ARA-TGG-TCA-TGT-GTG-GCG-G-3′ and RdRp_rev 5′-CAR-ATG-TTA-AAS-ACA-CTA-TTA-GCA-TA-C-3′. Probe was 5′-CAG-GTG-GAA-/ZEN/CCT-CAT-CAG-GAG-ATG-C-3′ (Label: FAM/IBFQ Iowa Black FQ). As positive control a specific target for E and RdRp gen of SARS-CoV2 was used and made by Integrated DNA Technologies. Control: 5′-TAA-TAC-GAC-TCA-CTA-TAG-GGT-ATT-GAG-TGA-AAT-GGT-CAT-GTG-TGG-CGG-TTC-ACT-ATA-TGT-TAA-ACC-AGG-TGG-AAC-CTC-ATC-AGG-AGA-TGC-CAC-AAC-TGC-TTA-TGC-TAA-TAG-TGT-TTT-TAA-CAT-TTG-GAA-GAG-ACA-GGT-ACG-TTA-ATA-GTT-AAT-AGC-GTA-CTT-CTT-TTT-CTT-GCT-TTC-GTG-GTA-TTC-TTG-CTA-GTT-ACA-CTA-GCC-ATC-CTT-ACT-GCG-CTT-CGA-TTG-TGT-GCG-TAC-TGC-TGC-AAT-ATT-GTT-3′.

### 4.7. Microscopy

For immunostaining, infected Calu-3 cells were fixed with 4% PFA for 15 min. Following a washing step with PBS, cells were blocked and permeabilized overnight with 1% BSA in PBS (+0.2% Triton) and afterwards stained with a polyclonal rabbit anti-NP antibody (GeneTex, Cat. No. GTX135357, Irvine, CA, USA) for 24 h. Subsequently, cells were incubated for 1 h with a goat anti-rabbit-AlexaFlour488 (Invitrogen, Cat. No. A11008, Waltham, MA, USA) and finally stained with 4′,6-Diamidino-2-phenyl-indol –dihydrochlorid (DAPI) (Sigma Aldrich, D9542, St. Louis, MO, USA) for 10 min. For analysis, immunostaining was quantitative analyzed with a PerkinElmer VictorX4 reader (488 nm) (PerkinElmer, Waltham, MA, USA) and pictures were taken using a CTL-ELISPOT reader (Cellular Technology Ltd., Shaker Heights, OH, USA).

### 4.8. Software and Statistics

GraphPad Prism 8.0 was used for statistical analyses and to generate graphs. Figures were generated with CorelDrawX7. To determine the combinatory effects of the treatment with PAM/Pio, the open-source and free web application SynergyFinder was used [42], and the drug interactions were analyzed by the commonly used Bliss independence [43].

## 5. Patents

Kinarus Therapeutics AG has filed a PCT and EP patent entitled “Methods of preventing or treating COVID-19 and related viral diseases or disorders” claiming the priority date of 11 August 2020.

## Figures and Tables

**Table 1:** IC_50_ and IC_90_ values of PAM and Pio against SARS-CoV-2 Wuhan type and all VoCs in Calu-3 cells.

	PAM		Pio	
	IC_50_	IC_90_	IC_50_	IC_90_
**Wuhan Type**	≈100 nM	≈3 µM	≈800 nM	≈10 µM
**Alpha**	≈250 nM	≈3 µM	≈800 nM	≈15 µM
**Beta**	≈250 nM	≈3 µM	≈900 nM	≈15 µM
**Gamma**	≈250 nM	≈3 µM	≈700 nM	≈15 µM
**Delta**	≈250 nM	≈4 µM	≈500 nM	≈12 µM
**Omicron**	≈250 nM	≈3 µM	≈700 nM	≈12 µM

## Data Availability

Data are included in the article.

## Supplementary Materials

The following supporting information can be downloaded at: https://www.mdpi.com/article/10.3390/ijms23126830/s1.
