# Supplementary material for: Synergistic Antiviral Activity of Pamapimod and Pioglitazone against SARS-CoV-2 and Its Variants of Concern
Source: Int J Mol Sci. 2022 Jun 20;23(12):6830. doi: 10.3390/ijms23126830 (PMC9224751; doi:10.3390/ijms23126830)
Supplement: Supplementary file 1 [file ijms-23-06830-s001.zip › ijms-1735847-supplementary.pdf]

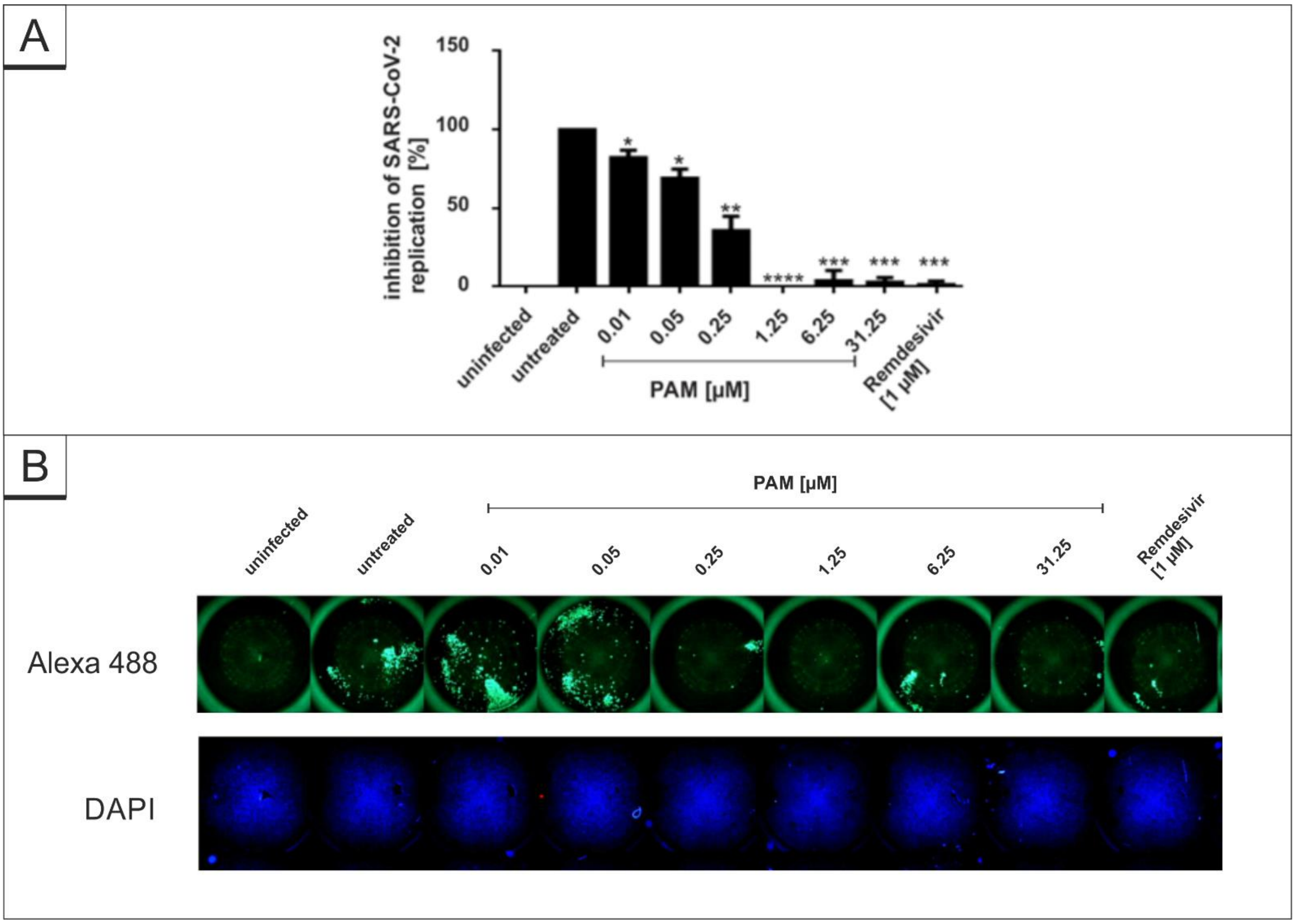

Figure S1. PAM inhibits SARS-CoV-2 replication in Calu-3 cells. Quantitative immunofluorescent microscopy was performed to detect the viral nucleocapsid protein in Calu-3 cells 30 hrs post-infection with SARS-CoV-2 Wuhan type using an Alexa488-conjugated anti-SARS-CoV-2 nucleoprotein (NP)-antibody or DAPI as a control. (**A**) Inhibition of SARS-CoV-2 replication (normalized fluorescent intensity (Alexa488/DAPI)) in cells treated with increasing concentrations of PAM. For calculation of % inhibition of SARS-CoV-2 replication, NP-staining was normalized to DAPI staining; each value is compared to the untreated control. Results show mean values of three independent experiments ± standard deviation. * $p \leq 0.05$, ** $p < 0.005$, *** $p < 0.0003$ and **** $p < 0.0001$ using an one-sample t-test. (**B**) Representative fluorescent images for each condition.
